# Critical older trauma patients

**DOI:** 10.1186/cc11059

**Published:** 2012-03-20

**Authors:** M Irazábal, S Yus, L Fernández

**Affiliations:** 1 Hospital La Paz, Madrid, Spain

## Introduction

The aim of this study was characterize the older injured patient in our setting and identify risk factors that might predict mortality. Trauma is the fifth leading cause of death over the age of 65. In Spain, it has become a major public health problem as a result of the increase of this population. It represents 30% of the trauma admissions to our ICU. Geriatric patients may have comorbidities, limited physiologic reserve, may be taking chronic medication and the injury pattern is different [1].

## Methods

We retrospectively analyzed trauma patients aged 65 years and older admitted to our ICU from January 2000 through December 2010. Three groups were formed on the basis of age: 65 to 70, 71 to 78 and older than 78 years. The Injury Severity Score (ISS) was categorized into three ranges: >12, 12 to 18 and >18. Variables studied include: age, gender, mechanism of injury, anticoagulant therapy (ACT), ISS, Glasgow Coma Scale (GCS) or presence of pupillary abnormalities and need for emergent neurosurgery (ENS) at admission. Primary outcome measures were in-hospital mortality and time to death. The secondary endpoint was to identify the effect of chronic medication on mortality. Categorical variables were compared by chi-squared test and continuous variables by Student's *t*/Mann-Whitney tests. Multiple logistic regression analysis was used to predict mortality. *P *< 0.05 was considered statistically significant.

## Results

The inclusion criteria were met by 261 patients. Age average was 75.57 years (SD 5.7). Male gender was more prevalent (58.5%) for all age groups. The median ISS was 17. The most frequent trauma mechanism was low-energy type (58.2%). Patients with chronic ACT numbered 41 (15.7%). The mean ICU stay was 12.8 days (SD 2.8). Global mortality was 34.1%. Age >78 years and ISS >18 were predictive of mortality (*P *< 0.05) with a HR of 6.0 (CI 2.5 to 14.6) and 1.01 (CI 1.01 to 1.05) respectively. Furthermore, the time to death was found to be earlier in both of the latter groups (*P *< 0.05). GCS <4 or bilateral mydriasis was associated with 100% mortality. About 15% of patients with low-energy trauma (LET) underwent ENS compared to 7.8% with high-energy trauma. For the same ISS category, ACT increases the risk with HR 2.7 (CI 1.2 to 6.3) of ENS compared with nonanticoagulated patients.

## Conclusion

LET accounted for most of the older trauma patients admitted to our ICU and had increased risk of death, especially with ACT. Although this is not necessarily secondary to alarming mechanisms.
